# The *tal* gene of lactococcal bacteriophage TP901-1 is involved in DNA release following host adsorption

**DOI:** 10.1128/aem.00694-24

**Published:** 2024-08-12

**Authors:** Sofía Ruiz-Cruz, Andrea Erazo Garzon, Christian Cambillau, Guillermo Ortiz Charneco, Gabriele Andrea Lugli, Marco Ventura, Jennifer Mahony, Douwe van Sinderen

**Affiliations:** 1 School of Microbiology & APC Microbiome Ireland, University College Cork, Cork, Ireland; 2 Laboratoire d’Ingénierie des Systèmes Macromoléculaires (LISM), Institut de Microbiologie, Bioénergies et Biotechnologie (IMM), Aix-Marseille Université—CNRS, Marseille, France; 3 Department of Chemistry, Life Sciences, and Environmental Sustainability, Laboratory of Probiogenomics, University of Parma, Parma, Italy; The Pennsylvania State University, University Park, Pennsylvania, USA

**Keywords:** *Lactococcus cremoris*, TP901-1 phage, Tal, DNA release, recombineering

## Abstract

Temperate P335 phage TP901-1 represents one of the best-characterized Gram-positive phages regarding its structure and host interactions. Following its reversible adsorption to the polysaccharidic side-chain of the cell wall polysaccharide of its host *Lactococcus cremoris* 3107, TP901-1 requires a glucosylated cell envelope moiety to trigger its genome delivery into the host cytoplasm. Here, we demonstrate that three distinct single amino acid substitutions in the Tal protein of TP901-1 baseplate are sufficient to overcome the TP901-1 resistance of three *L. cremoris* 3107 derivatives, whose resistance is due to impaired DNA release of the phage. All of these Tal alterations are located in the N-terminally located gp27-like domain of the protein, conserved in many tailed phages. AlphaFold2 predictions of the Tal mutant proteins suggest that these mutations favor conformational changes necessary to reposition the Tal fiber and thus facilitate release of the tape measure protein from the tail tube and subsequent DNA ejection in the absence of the trigger otherwise required for phage genome release.

**IMPORTANCE:**

Understanding the molecular mechanisms involved in phage-host interactions is essential to develop phage-based applications in the food and probiotic industries, yet also to reduce the risk of phage infections in fermentations. *Lactococcus*, extensively used in dairy fermentations, has been widely employed to unravel such interactions. Phage infection commences with the recognition of a suitable host followed by the release of its DNA into the bacterial cytoplasm. Details on this latter, irreversible step are still very scarce in lactococci and other Gram-positive bacteria. We demonstrate that a component of the baseplate of the lactococcal phage TP901-1, the tail-associated lysin (Tal), is involved in the DNA delivery into its host, *L. cremoris* 3107. Specifically, we have found that three amino acid changes in Tal appear to facilitate structural rearrangements in the baseplate necessary for the DNA release process, even in the absence of an otherwise required host trigger.

## INTRODUCTION

*Lactococcus lactis* and *Lactococcus cremoris* are among the most frequently used microorganisms in global dairy fermentations. Bacteriophages (phages), which are viruses that infect bacteria, represent a major economic concern to the dairy industry as they regularly delay or disrupt the fermentation process. Therefore, phages infecting *Lactococcus* have been extensively studied to investigate their prevalence, diversity, and host range-determining factors. All known lactococcal phages possess a tail, and 10 distinct groups have been distinguished based on their morphology and genetic relatedness (1). Members of the diverse P335 group are included among the most frequently encountered phages in dairy fermentations.

P335 group phages, which comprise both virulent and temperate phages (2), exhibit a narrow host range, which is believed to reflect a highly specific interaction with their cognate host (3). This interaction is mediated by the so-called “adhesion device,” which is located at the distal end of the phage tail, and is responsible for the recognition of, and reversible binding to, a suitable host-encoded receptor (3). The primary receptor for P335 phages, as well as the majority of lactococcal phages, is known to be (a component of) a specific cell wall polysaccharide (CWPS), which is composed of a peptidoglycan-embedded rhamnan and a surface-exposed oligo- or poly-saccharidic decoration or side-chain (4–6). Furthermore, these CWPS structures may contain additional sugar decorations due to the activity of three-component glycosylation systems (TGSs) (7). These TGSs typically consist of (i) a membrane-associated undecaprenyl-P-sugar (und-P) activating glycosyltransferase, (ii) a flippase that transports the Und-P-sugar moiety to the extracytoplasmic side of the membrane, and (iii) a polytopic GT that catalyzes attachment of the sugar to its final acceptor (8).

The P335 phage TP901-1 is one of the most thoroughly characterized Gram-positive phages and, along with Tuc2009, the best studied lactococcal phage with respect to their structural characteristics (9–11). The structure of the adhesion device or base plate of TP901-1 has previously been partially determined by electron microscopy (9, 12) and X-ray crystallography (11). Recently, AlphaFold2 (13) was used to further assess details of the base plate structure. It consists of six BppU (baseplate protein, upper component) trimers arranged around a central Dit (distal tail protein) hexamer. Each BppU trimer projects three RBP (receptor-binding protein) trimers (11, 14). At the distal extremity of the tail, a trimeric Tal (tail-associated lysin) protrudes, forming the core of the baseplate. It is known that TP901-1 Tal can undergo proteolytic processing, resulting in the removal of its C-terminal domain, which exhibits cell wall-degrading activity (15). The resulting heterogeneous phage population serves to facilitate the phage to infect bacteria most effectively where levels of cell wall cross-linkage may differ (15). The central channel of the tail is proposed to be filled by the TMP (tape measure protein) (14, 16).

TP901-1 recognizes and reversibly adsorbs to the polysaccharidic side-chain of the CWPS of *L. cremoris* 3107 via its RBPs. This CWPS side-chain (previously termed the polysaccharide pellicle or PSP) of the lactococcal 3107 strain is also the primary receptor of the P335 phage LC3 (17, 18). Following the RBP-mediated adsorption, the phage infection process continues with an irreversible event which involves phage DNA delivery into the bacterial cytoplasm. To date, information pertaining to this DNA delivery or release event in Gram-positive infecting phages remains scarce. Based on the characterization of three TP901-1-resistant derivatives of *L. cremoris* 3107, it has previously been proposed that TP901-1 and LC3 utilize distinct DNA release trigger(s) or different DNA entry pathways (18). Both phages were shown to be capable of adsorbing these lactococcal derivatives (18), whereas TP901-1 DNA release was blocked causing phage resistance (18, 19). Recently, we demonstrated through comparative genome analysis of these resistant derivatives and subsequent complementation experiments that two distinct GT-encoding genes from *L. cremoris* 3107 are required for TP901-1 DNA internalization (19). These two GTs form part of a TGS believed to be involved in glucosylation of an unknown cell envelope-associated moiety, which is currently proposed to represent the secondary receptor or molecular trigger for TP901-1 DNA release. The resistant derivative designated as E121 encodes a truncated GT, CsdC_3107_, which in the parent strain 3107 is the first GT of the system. Mutants E119 and E126 encode a truncated GT termed as CsdG_3107_, which in strain 3107 catalyzes the last step of the substrate glucosylation process (19). This is the first sugar decoration that has been implicated as a trigger of the DNA delivery step of a Gram-positive phage, prompting us to investigate the phage structure/s implicated in such interaction or process. In the current study, spontaneous TP901-1 mutants able to infect the E119, E121, and E126 mutant strains were isolated. Their comparative genome analysis shows that particular spontaneous mutations in the TP901-1 *tal* gene allow bypass of the DNA release trigger, which was corroborated by targeted mutational analysis. Finally, the effect of such mutations on Tal structure was predicted using AlphaFold2.

## MATERIALS AND METHODS

### Bacterial strains and growth conditions

*L. cremoris* 3107 (20) wild type (WT) and its derived TP901-1-resistant mutants E119, E121, and E126 (18) were grown at 30°C in M17 broth/agar (Oxoid, UK) supplemented with 0.5% glucose (GM17). *L. cremoris* NZ9000_TP901-1*erm* carrying TP901-1*erm* prophage and plasmid pJP005 (15) was grown at 30°C in GM17 supplemented with 2 µg mL^−1^ of Erythromycin (Ery) and 5 µg mL^−1^ of Chloramphenicol (Cm).

### Recombineering and oligonucleotides

All oligonucleotides used in this study are shown in Table 1. Recombineering was performed as previously described (15, 21, 22). Briefly, competent cells of *L. cremoris* NZ9000_TP901-1*erm* harboring pJP005 were transformed with 500 µg of the appropriate recombineering oligonucleotide, and after recovery, serial dilutions were plated on GM17 agar plates containing 2 µg mL^−1^ Ery and 5 µg mL^−1^ Cm. Colonies were screened by mismatch amplification mutation analysis-PCR (22), and those containing the desired mutation were further purified. Recombineering oligonucleotides were ordered from Integrated DNA Technologies (Belgium), whereas all other oligonucleotides were ordered from Eurofins Genomics (Germany).

**TABLE 1:** Oligonucleotides^*a*^

Oligo	Sequence (5′−3′)
218Rec	A*A*A*T*T*CAATTCTTCGCCCATAACCGTCACCAACTTCTTCTACTTTGCCCCTTCCAATTAGGGCAGTAACAATATTTGTACGGTCTTGTTG
226Rec	T*T*A*C*C*ATTTGACTTTCTCCACTCGACATCTGAAAATTCGATGCGCCTTCTATAACCGTCACCAACTTCTTCACCCTTCCCACGTCCAA
381Rec	C*T*C*G*A*ATGATATCAGATTGATACTTCCCAATTTCTGTACTATCGTAGCGCGTCATTTTATTATTATCAAGGTCAGAGATATTGCTTTG
603Rec	T*G*A*C*T*TCTGGTGGGTATTGACCATTCCAACCTGTATAGATCTAGCCAGAGTTTCCGCCACCATTAGTATCAATTTTTGTT
381ScFw	GGTGACGGTTATGGGCGAAGA
381ScRv	GAATTGCATTAATCCCTTGGAG
381MAMA	CCAATTTCTGTACTATCGTAGCGC
200ScFw	GATGAATGACACACTTTCAG
200ScRv	GCTTTGAACTTGTGACAGTTG
218MAMA	CCAACTTCTTCTACTTTGCCCCT
226MAMA	CTGAAAATTCGATGCGCCTTCT
603ScFw	ATTGAAGAATCTGATCATTTTCTTGTAGC
603ScRv	TTGGAGCAATATAACCTCCGCC
603MAMA	CAACCTGTGTCTCCATCTCCACTA

^*a*^ Asterisk denotes phosphorothioate linkages of recombineering oligonucleotides.

### Bacteriophage assays and isolation of TP901-1*erm* escape mutants

TP901-1*erm*, a derivative of phage TP901-1 harboring an Ery resistance marker (23), was used for all assays. TP901-1*erm* prophage was induced from its lysogenic host *L. cremoris* NZ9000_TP901-1*erm* when it reached an optical density at 600 nm (OD_600nm_) of approximately 0.2, using 0.5 µg mL^−1^ mitomycin C (MitC). The TP901-1*erm*-derived prophages generated by recombineering were induced in the same way. Where necessary, phages were propagated in 10 mL of GM17 broth cultured with *L. cremoris* 3107 (or the relevant mutant) at an approximate OD_600nm_ of 0.2. Either plaques, 100–200 µL of a lysate or the resulting lysate of MitC induction were used for propagation. SM buffer (10 mM CaCl_2_, 100 mM NaCl, 10 mM MgSO_4_, and 50 mM Tris-HCl at pH 7.5) was employed as the diluent in all phage assays.

Spontaneous TP901-1*erm* escape mutants (capable of infecting *L. cremoris* 3107 E-derivatives) were isolated from standard plaque assays (24). Specifically, 200-µL aliquots from overnight propagations of each *L. cremoris* 3107 E-derivative (E119, E121, and E126) were mixed with 10^8-9^ PFU of TP901-1*erm* from a fresh propagation, and this mixture was plated as described (24) and incubated at 30°C overnight. This procedure was independently performed four times in order to increase the likelihood of isolating plaques arising from independent events. Isolated visible plaques were propagated once or twice on the specific E-mutant used for the bacterial lawn. The efficiency of plating (EOP) was determined as the ratio of a specific phage titer on a *L. cremoris* 3107 E-derivative strain relative to that of the parent strain *L. cremoris* 3107 WT.

Lysogenization assays were performed as previously described (19). The required strain was grown until an approximate OD_600nm_ of 0.2 was reached. Equal volumes of phages and bacteria were mixed at an MOI of 0.05 and incubated at 30°C for 1 hour to allow one round of infection to occur. Following incubation, cells were diluted as necessary and plated on GM17 with and without 2 µg mL^−1^ Ery. Plates were incubated anaerobically for 48 hours at 30°C. The frequency of lysogenization was determined by calculating the total number of obtained Ery resistant lysogens per mL, divided by the total colony forming units per ml (CFU mL^−1^) obtained.

### Phage DNA extraction, genome sequencing, assembly, and bioinformatic analysis

Phage genomic DNA was extracted from the isolated TP901-1*erm* escape mutants and TP901-1*erm* WT using a Phage DNA Isolation Kit (Norgen Biotek, Canada). Moreover, TP901-1*erm* WT genomic DNA was extracted and sequenced in each batch of experiments. Phage genomic DNA sequencing was performed using an Illumina MiSeq Sequencing System. Genome assemblies were performed with SPAdes v3.14.0 via the MEGAnnotator2 pipeline (25). Open reading frame (ORF) prediction was performed with Prodigal v2.6 (26), and automatic ORF annotation was performed with DIAMOND against NCBI RefSeq database and INTERPRO against PFAM database (27). Subsequently, comparative genome and single nucleotide polymorphism (SNP) analysis was performed between the phage genome sequence of TP901-1*erm* escape mutants and that of TP901-1*erm* WT to identify mutations that may have caused these mutant phages to overcome the TP901-1 phage resistance of 3107 mutant strains E119, E121, and E126. Bowtie2 alignment (28), followed by the application of SAMtools (29) to extract base variants, was employed.

### Statistical analysis

Statistical analyses were performed using JASP software version (0.18.3) (30). For evaluation of differences in the lysogenization frequency and the EOPs of phages at different temperatures, first, we performed the Shapiro-Wilk test to assess whether the data were normally distributed and the Levene’s test to test the homogeneity of variances. If the data were normally distributed and the variances were equal, we performed one-way analysis of variance (ANOVA), followed by Tukey’s test. If not, we conducted the Kruskal-Wallis test followed by Dunn’s test. In all cases, differences are considered significant when the *P* value < 0.05.

### AlphaFold2 predictions and analysis

AlphaFold2 (13) predictions were performed using either a Colab notebook running AlphaFold v2.3.1 (https://colab.research.google.com/github/deepmind/alphafold/blob/main/notebooks/AlphaFold.ipynb) or HPC resources from GENCI-IDRIS running AlphaFold v2.3.1. The pLDDT values of any predicted structure, stored in the pdb file as B-factors, as well as the PAE, were plotted and are shown in Fig. S1. The final predicted protein or domain structures were submitted to the Dali server (31) to identify the closest structural homologs in the PDB. Dali provides a root mean square deviation value in Å, as well as an aggregated factor called Z-value. A Z-score above 20 means the two structures are definitely homologous, between 8 and 20 means the two are probably homologous, between 2 and 8 is a gray area, and a Z-Score below 2 is not significant. Visual representations of the structures were prepared with ChimeraX (32). Coot (33) was used to assemble predictions and visually analyze the predictions.

## RESULTS

### Isolation of TP901-1 escape mutants able to overcome the resistance of *L. cremoris* 3107 E-derivatives

Three distinct TP901-1-resistant (but LC3-sensitive) mutants of *L. cremoris* 3107, named E119, E121, and E126, had previously been isolated following chemical mutagenesis with ethyl methanesulfonate (18). We recently demonstrated that the resistance phenotype observed in those derivatives is due to the presence of mutations within two distinct GT-encoding genes, which form part of an *L. cremoris* 3107 TGS, involved in the glucosylation of a cell-envelope moiety, the latter believed to be involved in triggering TP901-1 DNA release (19). In all three TP901-1-resistant mutants, the mutations led to the introduction of a premature stop codon and, therefore, non-functional truncated products (19). Mutant E121 carries an insertion within *csdC_3107_*, which encodes a GT expected to catalyze the transfer of glucose (Glc) from uracil-diphosphate glucose (UDP-Glc) to Und-P, as shown by heterologous enzyme complementation (19). Mutants E119 and E126 each carry a distinct mutation within *csdG_3107_*, predicted to encode a GT that transfers this glucose to the final cell-envelope acceptor (19).

Since our attempts to identify the suspected glucosylated cell envelope-associated moiety have so far been unsuccessful (19, 34), we decided to investigate whether it was possible to isolate TP901-1 escape mutants capable of overcoming the phage-resistance of E121, E119, and E126 derivatives. To this end, 10^8-9^ PFU of TP901-1*erm* WT was mixed with grown cultures of each of the three *L. cremoris* 3107 E-mutants, after which plaque assays were performed. This procedure was independently executed four times, using a freshly prepared lysate of TP901-1*erm* WT. Escape mutants were observed at a very low frequency (usually less than five plaques per attempt representing an approximate frequency of <5 × 10^−8^), albeit slightly more frequently on the *L. cremoris* 3107 E119 strain relative to E121 and E126. Plaques were propagated once or twice on growing liquid cultures of the corresponding lactococcal mutant used to isolate them, and the presumed mutant phage lysate was tested by standard spot assays using *L. cremoris* 3107 and the three TP901-1-resistant E-derivatives. However, either the phages present in some of the visible plaques failed to propagate or the phage lysate did not produce discernible zones of lysis on the lawns formed by any of the strains tested. At the same time, apparent *bona fide* TP901-1*erm* escape mutants produced clearing in lawns of *L. cremoris* 3107 WT and the three E-derivatives, irrespective of the mutant strain used to isolate them. This finding indicates that these TP901-1*erm* phage mutants do not require the presence of the glucosylated moiety on the cell surface of the host to release their DNA. TP901-1*erm* mutants were named after the strain used to isolate them followed by a letter, in alphabetical order of isolation. A total of 14 genetically distinct escape mutants were isolated (see Table 2 and section below): 8 were isolated on E119, 3 on E121, and 3 on E126.

**TABLE 2:** Total number of SNPs found in each spontaneous mutant^*e*^^,^ ^*f*^

TP901-1*erm* spontaneous mutant	Number of SNPs	SNP position in *tal*	Nucleotide change			Amino acid change	Location/domain
E119A	174	653	G	to	C	G218A	Structural domain
E119B/ E121B^*a*^/ E121C^*b*^	13/1/1	653	G	to	T	G218V	Structural domain
E119A	174	654–56	Deletion			E219del	Structural domain
E119D^*b*^/ E119E/E119F^*b*^^, *c*^/E119G^*d*^	1/3/1/136	676	G	to	A	G226R	Structural domain
E119C/ E121A/E126A/E126B/E126C	76/43/74/109/87	1,141	T	to	C	W381R	Structural domain
E119H^*b*^	1	1,809	G	to	A	G603D	Proteolytic processing site

^*a*^ Only one SNP but query coverage 92%.

^*b*^ Only one SNP.

^*c*^ E119F is genetically identical to E119D, but it was included in the table because it was recovered in a different batch of experiments.

^*d*^ E119G has 94 mutations in *tal* that results in 20 aa changes.

^*e*^ Position and nucleotide change found in the *tal* gene, as well as the resulting mutation in the encoded aa and its domain location.

^*f*^ El19I has no mutations in *tal*. Its genome contains 21 SNPs, which results in 3 aa changes in the terminase large subunit (TerL), 8 aa substitutions in the tail-acting protein (Tap), and 1 aa change in tail terminator protein (Ttp).

### Most TP901-1*erm* escape mutants exhibit a single missense substitution in Tal

The genomes of TP901-1*erm* escape mutants as well as the genome of TP901-1*erm* WT were sequenced to identify the mutated gene(s) that is (are) responsible for allowing the phage to overcome the phage resistance phenotype. The genome of TP901-1 WT is 37.6 kb in length, has a 35.38% G + C content, and carries 56 predicted protein-coding regions (35). Following comparative genome analysis between the Illumina-sequenced TP901-1*erm* WT and phage derivatives, major deletion or insertion events were not identified. Therefore, a SNP analysis was performed to identify mutation events that may have caused the TP901-1*erm* escape mutants to overcome phage resistance. A SNP analysis was performed using the published TP901-1 data and the Illumina TP901-1*erm* WT data to rule out sequencing errors. To discard SNPs not involved in TP901-1 DNA release, TP901-1*erm* escape mutants were compared with the genomic sequence of the TP901-1*erm* WT obtained from the lysate used to isolate them. Overall, 15 TP901-1*erm*-independent escape mutants (Table 2) were obtained, of which two, i.e., mutants E119D and E119F, were shown to be genetically identical, though obtained in two separate attempts (in total, 14 genetically distinct mutants). Furthermore, several mutant phages were excluded as they were isolated in the same batch of experiments and their genome sequence turned out to be genetically identical and thus were considered to have originated from a single mutant. The genomes of the TP901-1*erm* escape mutants exhibit similar characteristics to that of the parent phage. Using as threshold an allelic variation frequency of 80%, the number of SNPs identified on the spontaneous phage mutants ranged between 1 and 174 SNPs (Table 2). Some of these SNPs occurred in more than one of the TP901-1*erm* escape mutant genomes and with some genes containing more than one mutation in a given genome. Interestingly, the 2,757-bp gene annotated as *tal* (tail-associated lysin/ORF47), which encodes a 918 amino acid (aa) product, was mutated in 14 out of the 15 TP901-1*erm* mutant genomes (Table 2). Among the escape mutants, E119A and E119G contain more than one mutation in *tal*, while the remaining mutants were shown to carry a single missense substitution. Remarkably, five of the phage mutants were shown to carry just a single SNP within their genomes: in mutants E119D/F, this mutation causes a glycine (G) to valine (V) substitution at aa residue 218 of Tal; the SNP in mutants E121B/C brings about a G to arginine (R) change at aa position 226 of Tal, while the SNP in mutant E119H produces a G to aspartic acid (D) alteration at aa position 603 of Tal. G218V and G226R substitutions also appear in other spontaneous TP901-1*erm* mutants, being the more frequently found mutations in Tal, along with a tryptophan (W) substitution to R at aa position 381 of the Tal protein. To determine how efficiently TP901-1*erm* spontaneous mutants are able to infect *L. cremoris* 3107 E-derivatives, quantitative plaque assays were performed using representative mutant phages possessing one of the most frequently obtained mutations (G218V, G226R, and W381R) or the substitution G603D (Table 3). Additionally, two phages containing the same mutation in *tal* were tested, i.e., E119B and E121C, to analyze whether having one SNP or more, and the strain used to isolate them, had any effect on the EOP. Interestingly, each phage mutant tested exhibited a higher EOP on *L. cremoris* 3107 E119 when compared with that of E121 and E126. Mutant phages E119B and E121C showed similar EOPs on each host, while E119H exhibited the lowest EOP on all E-derivatives (Table 3). Phages E119B, E121C, and E119F produced smaller plaques on the E-mutants than on *L. cremoris* 3107 WT, being in some cases pinpoint plaques. The ability of these mutants to overcome the phage insensitivity of *L. cremoris* 3107 E119, E121, and E126 mutants suggests that either Tal is interacting with the cell envelope component glucosylated by the TGS CsdCG_3107_ from *L. cremoris* 3107 or TP901-1 escape mutants bypass the need for this trigger to “activate” Tal.

**TABLE 3:** EOPs^*a*^ of representative spontaneous TP901-1*erm* mutants on *L. cremoris* 3107 E-derivatives

Phage	*L. cremoris* 3107 E-derivatives		
	E119	E121	E126
TP901-1*erm*	≤10^−8^	≤10^−8^	≤10^−8^
E119B (Mut G218V)	0.14 ± 0.03	0.10 ± 0.03	0.06 ± 0.03
E121C (Mut G218V)	0.14 ± 0.02	0.07 ± 0.03	0.07 ± 0.06
E119F (Mut G226R)	0.14 ± 0.09	0.07 ± 0.04	0.01 ± 4.7 x 10^−3^
E119D (Mut W381R)	0.08 ± 0.02	0.08 ± 0.01	0.05 ± 0.01
E119H (Mut G603D)	0.03 ± 0.01	4.1 × 10^−4^ ± 7.0 x 10^−5^	8.7 × 10^−4^ ± 5.0 x 10^−4^

^*a*^ The EOP was determined as the ratio of a specific phage titer on a derivative strain to that of the parent strain *L. cremoris* 3107 WT.

### Three single amino acid substitutions in Tal are sufficient to overcome TP901-1 resistance of *L. cremoris* 3107 E-derivatives

The structure of TP901-1 Tal (residues 1–918) was predicted by AlphaFold2 and was assembled within the Dit/BppU/RBP heteromeric protein complex to complete the adhesion device structure (3, 14). The TP901-1 Tal N-terminus possesses a T4 phage gp27 fold (36), followed by two linker domains (residues 401–589) and two catalytic domains (residues 618–918). The first catalytic domain (residues 618–772) resembles a domain of a cell wall-degrading enzyme of *Bacillus* phage phi29, structurally related to lysozymes (3, 37), while the second one (residues 782–918) is a D,D-endopeptidase domain, shown to be able to hydrolyze peptidoglycan cross-bridges (15). To corroborate our indications that certain TP901-1 Tal alterations are essential to allow infection of *L. cremoris* 3107 E-derivatives, four specific mutations were incorporated in *tal* of TP901-1*erm* prophage by recombineering (22). These phages produce a Tal protein carrying the following single aa substitutions, which are also found in the spontaneous TP901-1*erm* escape mutants: G218V, G226R, W381R, and G603D (Table 2). The first three mutations are located within the T4 gp27-like N-terminal domain. The last mentioned (G603D) mutation is located in a Glycine-rich motif (NGGGNSGGGD; the first and last G of this sequence corresponding to G597 and G604, respectively) located before the first catalytic domain (16, 38). This motif can undergo proteolytic processing, resulting in a heterogeneous population of two phage types (15). It has been shown that a mutant TP901-1*erm* phage, TP901-1*erm*_Gly>Arg_, engineered to contain three R instead of G residues in the proteolytic site of Tal, is unable to undergo proteolysis and therefore possesses a full-length tail fiber. This phage was shown to be better adapted to efficiently infect cells with a higher degree of cross-linkage in their cell wall, such as cells in the stationary phase (15). Since we expected a similar effect with the Tal G603D mutation, TP901-1*erm*_Gly>Arg_ was also tested in our experiments.

First, to analyze if any of the engineered mutations affect the phage’s ability to infect the *L. cremoris* 3107 WT strain, following prophage induction from the lysogenic host and propagation on *L. cremoris* 3107, the titer of each mutant phage was determined on *L. cremoris* 3107 (Table 4). Lysates resulting from inductions contained 1–2 × 10^7^ PFU mL^−1^, except for TP901-1*erm*_W381R_, which produced a lysate containing 1 × 10^6^ PFU mL^−1^. The propagations were performed in parallel, under the same conditions, using 100 µL of each mutant “induction” per 10 mL of culture of *L. cremoris* 3107. All phages increased their initial titer by at least 10-fold, indicating that all are able to produce new infective virions (Table 4), although TP901-1*erm*_G218V_ seemed to produce a smaller number of infective virions compared with the other phages.

**TABLE 4:** Engineered TP901-1 mutants after propagation on *L. cremoris* 3107 and EOP on *L. cremoris* 3107 E-derivatives

	Titer (PFU mL^−1^)	EOP *L. cremoris* 3107 E-derivative		
Phage	*L. cremoris* 3107	E119	E121	E126
TP901-1*erm*	2.4 × 10^8^ ± 1.7 × 10^8^	≤10^−8^	≤10^–8^	≤10^–8^
TP901-1*erm*_G218V_	2.6 × 10^6^ ± 3.6 × 10^5^	1.5 × 10^−2^ ± 7.0 × 10^−3^	6.4 × 10^−3^ ± 2.7 × 10^−3^	1.1 × 10^−3^ ± 8.1 × 10^−4^
TP901-1*erm***_G_**_22_**_6R_**	5.6 × 10^7^ ± 4.3 × 10^7^	1.0 × 10^−1^ ± 5.4 × 10^−2^	5.9 × 10^−2^ ± 3.8 × 10^−2^	9.8 × 10^−3^ ± 7.5 × 10^−3^
TP901-1*erm*_W381R_	1.6 × 10^6^ ± 1.1 × 10^6^	1.5 × 10^−1^ ± 5.0 × 10^−2^	1.1 × 10^−1^ ± 4.2 × 10^−2^	2.0 × 10^−2^ ± 1.1 × 10^−2^
TP901-1*erm*_G603D_	1.1 × 10^8^ ± 3.1 × 10^7^	N.D.^*a*^	N.D.	N.D.
TP901-1*erm*_GlyArg_	4.6 × 10^8^ ± 3.6 × 10^8^	N.D.	N.D.	N.D.

^*a*^ N.D., not determined.

Next, to investigate the effect of the mentioned Tal mutations on the ability of its corresponding mutant phages to infect lactococcal 3107-resistant derivatives, plaque assays were performed and their EOP was determined (Table 4). TP901-1*erm* mutants harboring mutations in the N-terminal domain of Tal (substitutions G218V, G226R, and W381R), formed visible plaques on each of the *L. cremoris* 3107 E-derivatives as well as on the WT strain, exhibiting the highest EOPs on E119 and the lowest EOP on E126. TP901-1*erm*_G226R_ and TP901-1*erm*_W381R_were shown to exhibit similar EOPs on the three lactococcal mutants, while TP901-1*erm*_G218V_ yielded EOPs around 10 times lower than the former two TP901-1_erm_ mutants. In all cases, plaques formed on the WT host had a regular size, while they were tiny on the three lactococcal E-derivatives, suggesting that these hosts could affect either the phage latent period or the burst size, apart from the rate constants for phage binding to the specific host (39). The reduction of plaque size on the E-derivatives was also observed with the spontaneous mutant phages E119B, E121C, and E119F, as mentioned above. Double phage mutants were also constructed by recombineering (TP901-1*erm*_G218V,W381R_ and TP901-1*erm*_G226R,W381R_). However, when they were induced from their lysogenic host by MitC, either the lysates were unable to infect *L. cremoris* 3107 WT and its derivatives, or no intact virions were formed. The other two single mutant phages tested, TP901-1*erm*_G603D_ and TP901-1*erm*_Gly>Arg_, seemed to overcome TP901-1 resistance of *L. cremoris* 3107 E-derivatives, but their EOP could not be accurately determined, as they produced very hazy plaques which were observed only after 48 hours of incubation. Plaque assays were also performed using an overlay containing agarose instead of agar, but no improvement in the plaque visualization was achieved. These findings reinforce the notion that TP901-1 Tal is implicated in the DNA release process triggered by the glucosylated moiety of *L. cremoris* 3107 and that three aa, G218, G226, and W381, and possibly G603, are involved in this interaction and/or in the genome delivery process.

**Fig 1:**
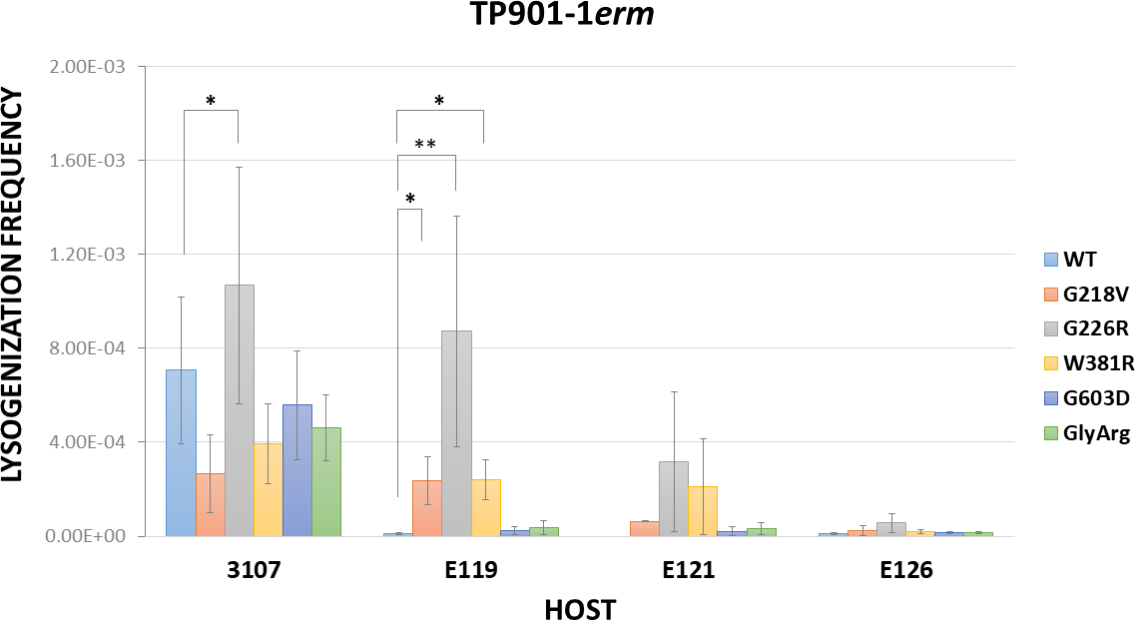
Engineered TP901-1*erm* mutant lysogenization frequency of *L. cremoris* 3107 and E-derivatives. Data are the mean of three biological replicates ± standard deviation. One-way ANOVA or Kruskal-Wallis test were performed to compare the lysogenization frequency of *L. cremoris* 3107, depending on the data distribution and homogeneity of variance. They were followed by Tukey’s test or Dunn’s test, respectively, to determine if there are significant differences compared with TP901-1*erm* WT. The lysogenization frequency of E121 was not statistically analyzed as TP901-1*erm* WT frequency was below the limits of detection of our assay ( Table S1). A *P* value ≤ 0.05 was considered significant and is represented by an asterisk “*”and a *P* value ≤ 0.01 was represented by two asterisks “∗∗.”

To support the previous results and considering that lysogeny assays reflect the capacity of a phage to adsorb to its host and to deliver its DNA followed by integration, rather than its ability to form a plaque, we determined the lysogenization frequencies of the engineered mutant phages. An MOI of 0.05 was used for these experiments since some of the phage mutants did not reach a high titer. TP901-1*erm* lysogenization frequencies of the three *L. cremoris* 3107 E-derivatives were lower than those of the WT strain (Fig. 1; Table S1). All tested engineered mutant phages exhibited a similar lysogenization frequency of *L. cremoris* 3107 (10^−4^), except for TP901-1*erm*_G226R_, whose lysogenization frequency was significantly higher than TP901-1*erm* (1.5-fold higher) (Fig. 1). The lysogenization frequencies obtained for *L. cremoris* E119 when tested using mutant phages harboring the Tal substitutions G218V, G226R, and W381R were significantly different compared with TP901-1*erm* WT and approximately the same frequency as of *L. cremoris* 3107 WT. The frequency of lysogenization of E119 with the other two phages was similar to TP901-1*erm* (Fig. 1). In the case of *L. cremoris* E121, a considerable increase in the frequency of lysogeny compared with TP901-1*erm* was observed with all phage mutants, but the statistical analysis was not performed as the frequency of lysogeny of TP901-1*erm* was below the detection limits (Table S1). The lysogenization frequencies of *L. cremoris* E119 and E121 by the phages carrying Tal substitutions G218V, G226, and W381R support the hypothesis that these three aa play an important role in Tal’s function. In contrast, *L. cremoris* E126 did not reveal any substantial increase in the frequency of lysogenization with any of the tested phages. Although this was unexpected, *L. cremoris* 3107 E-mutants carry additional mutations (19) which may affect growth and/or lysogeny establishment. Consistent with this, the EOP on *L. cremoris* E126 of most mutant phages (spontaneous or engineered; Tables 2 and 4) was lower compared with the EOP observed for the other two E-derivatives.

### TP901-1*erm*_W381R_ infectivity is temperature dependent

**Fig 2:**
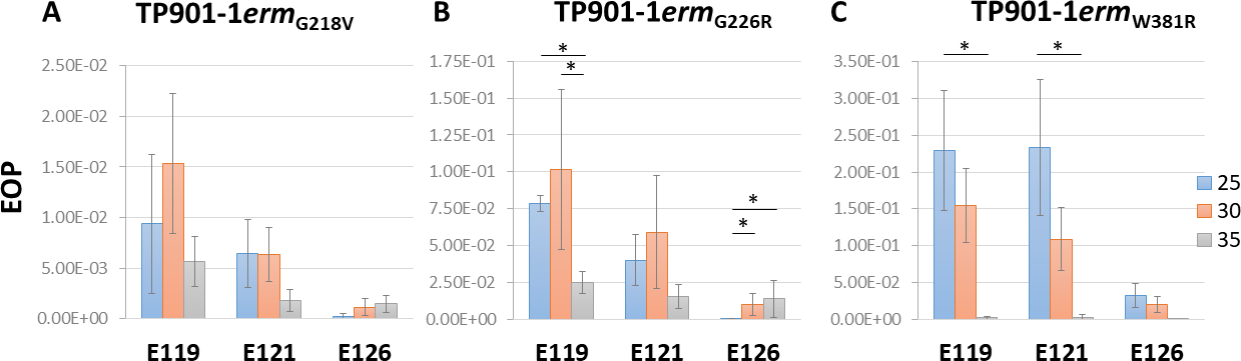
Engineered TP901-1*erm* mutants EOP on *L. cremoris* 3107 and E-derivatives at different temperatures. Data are the mean of three biological replicates ± standard deviation. One-way ANOVA or Kruskal-Wallis test were performed to compare the EOPs at different temperatures, depending on the data distribution and homogeneity of variance, followed by Tukey’s test or Dunn’s test, respectively, to determine differences between two temperatures. A *P* value ≤ 0.05 was considered significant and is represented by an asterisk “*.”

Temperature critically influences phage infection as it can affect host growth and its gene expression, the phage infection stages and type of cycle; and the phage *per se*, since temperature may affect the function of its structural components. We thus investigated the temperature effect on the infection of *L. cremoris* 3107 WT and E-derivatives by the engineered mutants. Lactococcal cells were grown at 30°C, while the plates were incubated overnight at the maximum temperature that allows growth of *L. cremoris* 3107, 35°C, as well as at a lower than optimal growth temperature for this strain, i.e., 25°C. First, we assessed that the titer of each phage mutant originating from the same lysate on *L. cremoris* 3107 WT plates incubated at the different temperatures was similar to the value obtained at 30°C. Then, we calculated the EOP values of three lactococcal E-derivatives (Fig. 2; Table S2). EOPs obtained at 30°C were also included in the table for comparison. TP901-1*erm*_G218V_ EOPs were similar on each mutant at each temperature (Fig. 2A; Table S2). TP901-1*erm*_G226R_ exhibited similar EOPs on E121 independently of the temperature used. EOP values on E119 were significantly different, exhibiting slightly higher EOPs when compared with 35°C. In contrast, on E126, the EOP significantly increased with temperature, being nearly 40-fold higher at 35°C compared with 25°C (Fig. 2B; Table S2). Regarding TP901-1*erm*_W381R_, the increase in temperature had the same effect on all mutants, and the EOPs on the three mutants decreased more than 100-fold as the temperature of incubation increased, being significantly different on E119 and E121 when compared with EOPs at 25°C to 35°C. Surprisingly, in both cases, the differences between 30°C and 35°C were not significant (Fig. 2C; Table S2). In general, the plaque size of TP901-1*erm*_G218V_ and TP901-1*erm*_G226R_ did not vary much with temperature. In the case of TP901-1*erm*_W381R_, plaques observed on E119 and E121 at 25°C were bigger compared with the other temperatures and had a similar size to the regular-size plaques observed on *L. cremoris* 3107 at 30°C.

### Modelling of Tal

**Fig 3:**
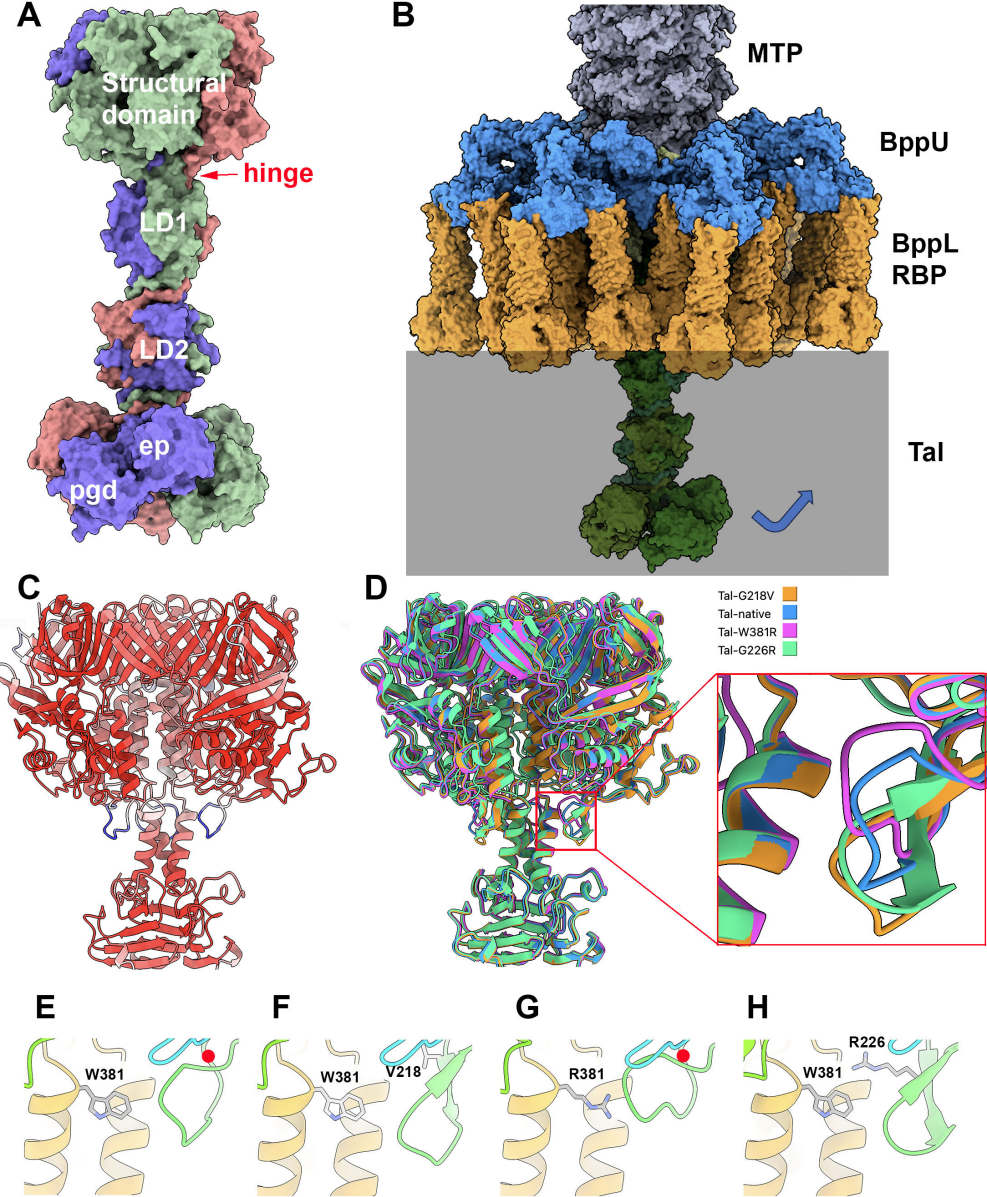
Structural analysis of TP901-1 Tal. (**A**) Surface representation of the structural prediction of trimeric Tal; structural domain (residues 1–380); LD1 and LD2, linker domains 1 and 2; pgd, peptidoglycan depolymerase domain; ep, endopeptidase domain. (**B**) Surface representation of the TP901-1 complete baseplate; MTP, major tail protein (gray); Dit, distal tail protein (yellow, hidden); BppU (blue); RBP/BppL (orange); Tal (green). The cell wall is symbolized by the gray area. (**C**) Ribbon representation of trimeric Tal N-terminus (1–463) colored by pLDDT. Note the flexible loops 217–227 in blue. (**D**) Superposition of native and mutated Tal [same orientation as (**C**)]; inset; close-up of helices 380–396 and loops 217–227 colored by monomer. (**E**) Ribbon and side-chain view of native Tal W381. (**F**) Ribbon and side-chain view of Tal W381 and mutant G218V. (**G**) Ribbon and side-chain view of W381R Tal. (**H**) Ribbon and side-chain view of Tal W381 and mutant G226R. (**E–H**) Ribbon is rainbow colored. (**E, G**) The red dots identify G218 and G226. Figure made with ChimeraX (32). The pLDDT values of any predicted structure, stored in the pdb file as B-factors, as well as the PAE were plotted and are shown in Fig. S1.

The release of AlphaFold2, a machine-learning, based model to predict protein structures developed by DeepMind, has represented a milestone advance in structural biology (40), due to its atomic accuracy even in cases in which no similar structure is known (13). It synergizes with experimental methods including X-ray crystallography and cryo-electron microscopy and enables structural analyses of large and flexible assemblies resistant to experimental approaches (41). The X-ray structure of the TP901-1 baseplate has previously been reported (PDB id 4v96), though without its Tal component (11). Recently, the full-length Tal trimer was predicted with AlphaFold2 (Fig. 3A) and added to the X-ray-determined structure of the baseplate (14) using Coot (33) (Fig. 3B). The central channel of Tal’s structural N-terminal domain (1–390) is filled by three α-helices that link this domain to the functional C-terminal domain. These helices and the C-terminal domain are believed to dramatically rearrange in order to allow TMP exit and subsequent DNA ejection in the early infection stages (14). Such a rearrangement has recently been reported for phage T5 (42, 43). Based on this, a similar mechanism is proposed to occur for TP901-1 upon baseplate/glucosylated moiety binding to allow consecutive TMP/DNA exit, involving the expulsion of the three helices within the Tal channel and rotation of Tal’s middle and C-terminal domain (Fig. 3B). These structural changes may be induced or facilitated by the impact of the Tal C-terminus on the cell wall and/or its peptidoglycan affinity due to the two cell wall-degradative enzymatic activities adjacently specified by this Tal portion (Fig. 3B). No saccharide-binding domains were found running Dali and Foldseek (31, 44) on linker domains, consistent with previous predictions (3). If such rotation and structural rearrangement occur in TP901-1 Tal, the rotation hinge should be located around residues 375–385 (Fig. 3A, C, and D). Mutations at positions 381, 226, and 218 may play an important role as W381 is located in the putative hinge area and faces G218 and G226. AlphaFold2 predictions of mutants G218V, G226R, and W381R suggest that a conformational change occurs in the loop 217–227 (Fig. 3E through H). These mutations may weaken the Tal rotation area and therefore favor the conformational changes necessary to reposition the Tal fiber and thus facilitate TMP/DNA ejection.

## DISCUSSION

Phage infection commences with an initial reversible adsorption to the host, followed by an irreversible step that coincides with DNA release into the bacterial cytoplasm. Knowledge on and understanding of this latter step in phages that infect Gram-positive bacteria is still very limited. It is known that *Lactococcus Ceduovirus* and TP901-1 require two distinct host receptors for reversible and irreversible adsorption, and in both cases, the primary receptor is part of the lactococcal CWPS. The Ceduovirus secondary receptor is the membrane‐associated protein YjaE (45, 46) or PIP (phage infection protein) (47, 48). It has been reported that the phage genes corresponding to l14-15-16 from phage c2 and ORF34-35-36 from phage bIL67 correlate to host lactococcal phage determinants Pip and YjaE, respectively (45). The annotation of the adhesion device of these phages is ambiguous in publicly available databases, but it has been suggested that l13-l14 represent functionally equivalent Dit-Tal components (3). Based on previous work, it has been suggested that TP901-1 irreversibly binds to an as yet unknown glucosylated cell envelope moiety, triggering DNA release (19). In the present study, we provide evidence to support the notion that the Tal protein of TP901-1, which forms part of its baseplate, is implicated in DNA release. Of note, the lactococcal-purified cell wall (without the plasma membrane) has been shown to be necessary and sufficient for adsorption and irreversible inactivation of (some) Skunaviruses (49).

**Fig 4:**
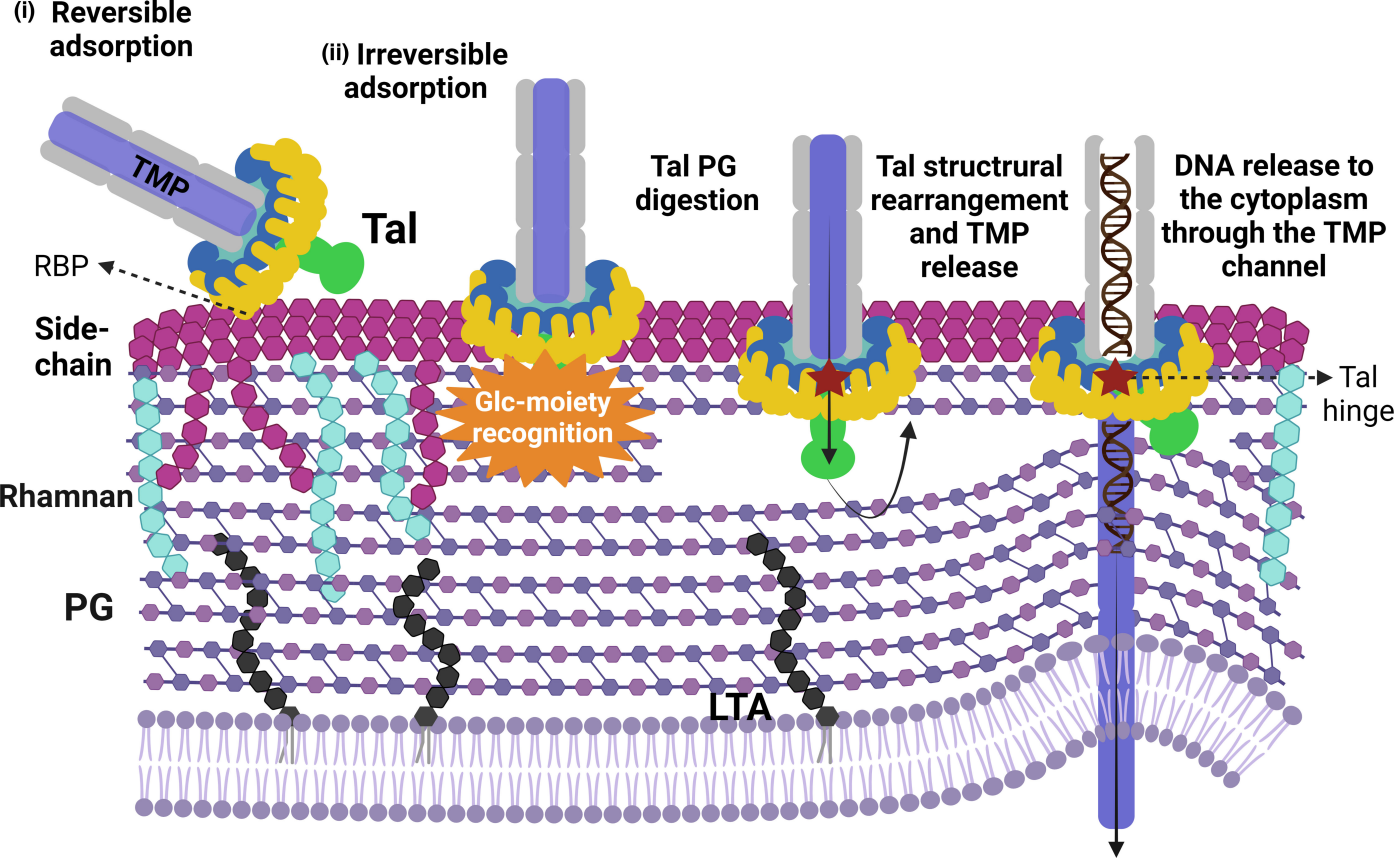
Schematic proposed model of TP901-1 first stages of infection. TP901-1 baseplate is depicted: MTP (gray); TMP (blue); Dit (turquoise); BppU (dark-blue); RBPs (yellow); Tal (green). The cell wall of *L. cremoris* 3107 WT contains a thick peptidoglycan (PG) layer; lipoteichoic acids (LTA) anchored to cytoplasmic membrane; CWPS, consisting of the rhamnan (light-blue) and the polysaccharidic side-chain (purple) and cell wall proteins (not shown). (i) TP901-1 recognizes and reversibly adsorbs to the side-chain of the CWPS of *L. cremoris* 3107 via its RBPs. (ii) TP901-1 irreversibly adsorbs to a glucosylated cell envelope-associated moiety, triggering conformational changes in Tal that facilitate release of the TMP from the tail tube, the formation of a channel, and the concomitant release of the genome into the cytoplasm. These structural changes may be induced or facilitated by the impact of the Tal C-terminus on the cell wall and its enzymatic activity, which may allow the membrane to “bulge” closer to this TMP channel. Tal mutations in the putative Tal rotation hinge or facing it, appear to weaken this Tal rotation area (red star), facilitating Tal rearrangements without the requirement of the glucosylated trigger. Created with BioRender.com.

Tal proteins from most phages are longer than ~400 residues and in some cases reach up to 2,000 residues or more. Typically, these proteins possess a conserved N-terminal structural domain, followed by an extension exhibiting large structural and functional diversity (3). The involvement of Tal in the early stages of infection has been experimentally demonstrated in other Gram-positive infecting phages. *Bacillus subtilis* phage SPP1, following recognition of glycosylated teichoic acids present on the host cell surface, irreversibly binds through Tal to the ectodomain of a membrane protein, YueB, an interaction that triggers genome delivery into the cytoplasm (50, 51). This interaction was shown by the isolation of phage mutants specifically affected in YueB binding and the use of antibodies against the C-terminal region of Tal, consisting of 1,108 residues, which interfered with its interaction with YueB and triggered DNA ejection (51). In the case of the *Listeria* phage A118, it has been shown that antibodies raised against gp18, the Tal protein, can neutralize adsorption of A118 (52). Tal protein from A118 possesses an extension of ~300 aa following the N-terminal structural domain, harboring a glycosidase domain at its extremity. Interestingly, the first 404 residues of SPP1 Tal fold as the A118 Tal N-terminal domain (53). Currently, we cannot affirm that TP901-1 Tal is directly interacting with the secondary receptor based on our results and structural predictions. Firstly, the mutations found here to be sufficient to overcome TP901-1 resistance are not part of a carbohydrate binding domain (CBM). In fact, no CBMs are predicted within Tal (3), although it is expected to possess peptidoglycan affinity due to its enzymatic activity (15). Nevertheless, it cannot be excluded that there may be, as yet, undiscovered alternative triggers for this process. Secondly, TP901-1 recognition of the secondary receptor seems to induce Tal conformational changes leading to TMP exit which is necessary for DNA release (Fig. 4). Tal mutations G218V, G226R, and W381R, which are either located in the putative Tal rotation hinge or facing it, appear to weaken this Tal rotation area, facilitating Tal rearrangements without the requirement of the glucosylated trigger. This mechanism is based on the one described for coliphage T5. Upon contact of the tail tip with T5’s receptor, the membrane protein FhuA, the Tal-like protein pb3, which obstructs the tail exit channel, opens and rotates on the tail side, thus allowing the TMP (pb2) to insert into the membrane (42, 43).

We have observed that the Tal mutations G226R and W381R have an effect on the EOPs at different temperatures (Fig. 2), although the effect of G226R is milder and contradictory depending on the host. The impact of temperature on phage infection can be attributable to different factors, i.e., the host gene expression or physical properties of the cell surface receptors (54, 55); the phage lifestyle (56) and its gene expression (57); and/or phage particle stiffness (58). Considering that our results show that the infectivity of only one of the mutants, TP901-1*erm*_W381R_, is greatly affected by temperature and shows the same tendency in the three E-derivatives, it seems that it is not a consequence of the aforementioned factors, as they may be expected to equally affect each mutant infection. Even though Tal substitutions G218V, G226R, and W381R are predicted to cause a similar structural impact, it could be that this specific mutation, W381R, leads to a temperature-sensitive phenotype. For instance, it has been described that some mutations in the tailspike-encoding gene of *Salmonella* phage P22 are critical for its proper folding when it is produced at restrictive temperatures. If the tail spike of such much mutants is formed at permissive temperature, they have similar thermal stability and biological activity properties to the wild type (59, 60).

The phenotypic effect of Tal mutation G603D is unclear. The spontaneous mutant harboring this single mutation formed plaques on the E-derivatives after overnight incubation (although it exhibited the lowest EOP compared with the other spontaneous mutants), whereas the recombineering mutant formed very hazy plaques after 48 hours. Given that the aa substitution is located in the Glycine-rich motif, in the junction between the second structural linker domain and the first catalytic domain, we presume that it cannot undergo proteolysis, as is the case for TP901-1*erm*_Gly>Arg_ (15). Therefore, the entire mutant population is expected to possess a full-length tail fiber, maintaining the cell wall-degrading domains, an adaptation that has been shown to promote TP901-1*erm*_Gly>Arg_ infection of stationary phase cells. The delay in the appearance of plaques is consistent with this hypothesis.

The latter mechanism for infection optimization under particular conditions was also described for the P335 phage Tuc2009 (15). The aa sequence identity of TP901-1 and Tuc2009 Tal’s is 96% (3, 15). In fact, as their baseplates are very similar, they have been classified in the same group (II) of adhesion devices stablished for P335 phages, that is based on comparative genome and morphological analyses (38). The main difference between the baseplates of TP901-1 and Tuc2009 is the presence of an additional protein termed BppA in the Tuc2009 baseplate (10, 11). Furthermore, their RBPs differ significantly, and accordingly, they target different lactococcal hosts (3, 38, 53). It would be interesting to define if, following initial attachment of Tuc2009 to its host *L. cremoris* UC509.9, DNA release to its host is similar to that observed for TP901-1, requiring the participation of Tal_Tuc2009_ and the presence of a glucosylated secondary receptor. Curiously, Tal_Tuc2009_ contains G218 and G226, while the residue 381 is a phenylalanine (F). This aa, together with G218 and G226, are also present in the putative Tal of an intact prophage found in the genome of *L. cremoris* 3107. The 3107 prophage is related to TP901-1 (93% nucleotide identity/47% coverage) (61). The putative Tal protein shares 97% and 95% aa identity with Tal_TP901-1_ and Tal_Tuc2009_, respectively. To our knowledge, it is not known if there is any interaction between TP901-1 and this prophage.

In the case of the P335 phage LC3, aa sequence identity between the LC3 RBP C-terminal domain and that of TP901-1 is 95%, with conserved receptor-binding residues, reflecting the fact that TP901-1 and LC3 are able to infect the same lactococcal strain (3, 38). However, their DNA release mechanism differ (18) and it has been suggested that this could be a consequence of different interactions with the host through their distinct adhesion devices (19). LC3 belongs to the group III of P335 phages, which encompass a “stubby” baseplate with a short Tal, the latter consisting of only the N-terminal structural domain (3, 38). Our results, which implicate TP901-1 Tal in the DNA release mechanism and the differences between both Tals, support the latter hypothesis of two distinct DNA release mechanisms.

It is well established that modular shuffling within phage structures, as happens with TP901-1, along with the diversity of host cell wall structures involved in phage sensitivity, results in a vast repertoire of possible phage-host combinations. However, it is still remarkable to note that a single aa substitution has such a profound effect on a complex, multi-stage process as phage infection, broadening the host range or completely changing phage susceptibility. This study has opened avenues for mutational analysis of the *tal* gene of TP901-1, as well as different phage assays with cell wall extracts. Moreover, the noteworthy effect of temperature on some phage mutants needs further detailed investigation in order to draw firm mechanistic conclusions. Indeed, shedding light on the molecular characteristics and precise function of the Tal protein is essential to understand the TP901-1 DNA release process. This demonstrates the value of acquiring a thorough understanding of the molecular mechanisms involved in phage-host interactions, as this information is essential not only to combat infections in undesirable contexts such as food fermentations but also to develop rationale phage-based applications such as therapeutic or biocontrol agents and biosensors.

## Data Availability

The Illumina raw reads of TP901-1erm mutants were deposited in SRA repository under BioProject no. PRJNA1097360.

## SUPPLEMENTAL MATERIAL

Supplemental figure and tables aem.00694-24-s0001.docx Fig. S1; Tables S1 and S2.

ASM does not own the copyrights to Supplemental Material that may be linked to, or accessed through, an article. The authors have granted ASM a non-exclusive, world-wide license to publish the Supplemental Material files. Please contact the corresponding author directly for reuse.
