# Supplementary material for: The tal gene of lactococcal bacteriophage TP901-1 is involved in DNA release following host adsorption
Source: Appl Environ Microbiol. 2024 Aug 12;90(9):e00694-24. doi: 10.1128/aem.00694-24 (PMC11409707; doi:10.1128/aem.00694-24)

**Table S1:** Engineered TP901-1 mutants lysogenization frequency of *L. cremoris* 3107 and E-derivatives

|  | ***L. cremoris* strain​** | | | |
| --- | --- | --- | --- | --- |
| **Phage** | **3107** | **E119** | **E121** | **E126** |
| **TP901-1*erm*** | 7.06 x 10^-4^ ± 3.10 x 10^-4^ | 1.08 x 10^-5^± 4.21 x 10^-6^ | <1 x 10^-6^ | 1.06 x 10^-5^± 3.09 x 10^-6^ |
| **TP901-1*erm*_G218V_** | 2.65 x 10^-4^ ± 1.65 x 10^-4^ | 2.35 x 10^-4^ ± 1.01 x 10^-4^ | 6.38 x 10^-5^ ± 1.64 x 10^-6^ | 2.37 x 10^-5^ ± 2.11 x 10^-5^ |
| **TP901-1*erm*_G226R_** | 1.07 x 10^-3^± 5.05 x 10^-4^ | 8.72 x 10^-4^ ± 4.91 x 10^-4^ | 3.17 x 10^-4^ ± 2.96 x 10^-5^ | 5.57 x 10^-5^ ± 3.98 x 10^-5^ |
| **TP901-1*erm*_W381R_** | 3.93 x 10^-4^ ± 1.69 x 10^-4^ | 2.42 x 10^-4^ ± 8.45 x 10^-5^ | 2.10 x 10^-4^ ± 2.03 x 10^-4^ | 1.80 x 10^-5^ ± 8.35 x 10^-6^ |
| **TP901-1*erm*_G603D_** | 5.57 x 10^-4^ ± 2.33 x 10^-4^ | 2.25 x 10^-5^± 1.80 x 10^-5^ | 2.09 x 10^-5^± 1.84 x 10^-5^ | 1.51 x 10^-5^± 2.81 x 10^-6^ |
| **TP901-1*erm*_GlyArg_** | 4.62 x 10^-4^ ± 1.42 x 10^-4^ | 3.76 x 10^-5^± 2.89 x 10^-5^ | 3.25 x 10^-5^± 2.51 x 10^-5^ | 1.65 x 10^-5^± 4.38 x 10^-6^ |

|  | ***L. cremoris* 3107 E-derivatives** | | | | | | | | |
| --- | --- | --- | --- | --- | --- | --- | --- | --- | --- |
|  | **E119** | | | **E121** | | | **E126** | | |
| **Temperature (°C)** | 25 | 30 | 35 | 25 | 30 | 35 | 25 | 30 | 35 |
| **TP901-1*erm*** | ≤10^-8^ | ≤10^-8^ | ≤10^-8^ | ≤10^-8^ | ≤10^-8^ | ≤10^-8^ | ≤10^-8^ | ≤10^-8^ | ≤10^-8^ |
| **TP901-1*erm*_G218V_** | 9.4 x 10^-3^± 6.9 10^-3^ | 1.5 x 10^-2^± 7.0 x 10^-3^ | 5.6 x 10^-3^± 2.5 x 10^-3^ | 6.4 x 10^-3^± 3.3 x 10^-3^ | 6.4 x 10^-3^ ± 2.7 x 10^-3^ | 1.8 x 10^-3^ ± 1.0 x 10^-3^ | 2.2 x 10^-4^ ± 3.2 10^-4^ | 1.1 x 10^-3^± 8.1 x 10^-4^ | 1.5 x 10^-3^± 8.4 x 10^-4^ |
| **TP901-1*erm*_G226R_** | 7.9 x 10^-2^± 5.5 x 10^-3^ | 1.0 x 10^-1^± 5.4 x 10^-2^ | 2.5 x 10^-2^± 7.2 x 10^-3^ | 4.0 x 10^-2^ ± 1.7 x 10^-2^ | 5.9 x 10^-2^± 3.8 x 10^-2^ | 1.6 x 10^-2^ ± 8.1 x 10^-3^ | 3.6 x 10^-4^± 1.7 x 10^-4^ | 9.8 x 10^-3^± 7.5 x 10^-3^ | 1.4 x 10^-2^± 1.2 x 10^-3^ |
| **TP901-1*erm*_W381R_** | 2.3 x 10^-1^± 8.1 x 10^-2^ | 1.5 x 10^-1^± 5.0 x 10^-2^ | 1.7 x 10^-3^ ± 2.6 x 10^-3^ | 2.3 x 10^-1^± 9.2 x 10^-2^ | 1.1 x 10^-1^± 4.2 x 10^-2^ | 2.7 x 10^-3^ ± 3.4 x 10^-3^ | 3.2 x 10^-2^± 1.6 x 10^-2^ | 2.0 x 10^-2^± 1.1 x 10^-2^ | 1.3 x 10^-4^ ± 1.7 x 10^-4^ |

**Table S2:** EOP on *L. cremoris* 3107 E-derivatives at 25, 30 and 35°C.

**Supplementary Figure S1**: AlphaFold2 predicted local distance difference test (pLDDT) and predicted aligned errors (PAE) of the native TP901-1 Tal and mutants (residues 1-463).
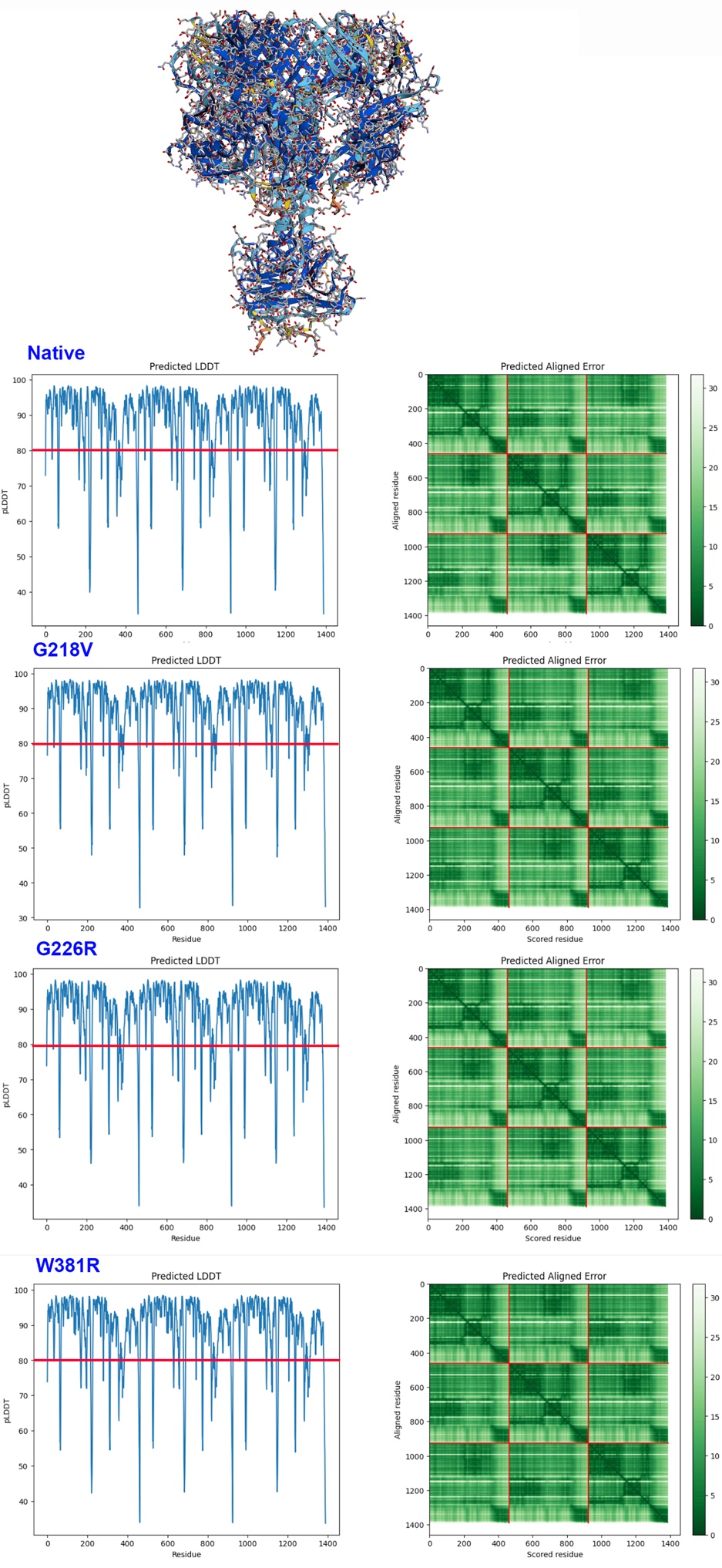

Supplement: Supplemental figure and tables — Fig. S1; Tables S1 and S2. [file aem.00694-24-s0001.docx]
